# Contrasting Effects of Land Use Intensity and Exotic Host Plants on the Specialization of Interactions in Plant-Herbivore Networks

**DOI:** 10.1371/journal.pone.0115606

**Published:** 2015-01-07

**Authors:** Walter Santos de Araújo, Marcos Costa Vieira, Thomas M. Lewinsohn, Mário Almeida-Neto

**Affiliations:** 1 Programa de Pós-Graduação em Ecologia e Evolução, Instituto de Ciências Biológicas, Universidade Federal de Goiás, Goiânia, Goiás, Brazil; 2 Laboratório de Interações Ecológicas e Biodiversidade, Departamento de Ecologia, Instituto de Ciências Biológicas, Universidade Federal de Goiás, Goiânia, Goiás, Brazil; 3 Laboratório de Interações Insetos-Plantas, Instituto de Biologia, Universidade Estadual de Campinas, Campinas, São Paulo, Brazil; Oklahoma State University, UNITED STATES

## Abstract

Human land use tends to decrease the diversity of native plant species and facilitate the invasion and establishment of exotic ones. Such changes in land use and plant community composition usually have negative impacts on the assemblages of native herbivorous insects. Highly specialized herbivores are expected to be especially sensitive to land use intensification and the presence of exotic plant species because they are neither capable of consuming alternative plant species of the native flora nor exotic plant species. Therefore, higher levels of land use intensity might reduce the proportion of highly specialized herbivores, which ultimately would lead to changes in the specialization of interactions in plant-herbivore networks. This study investigates the community-wide effects of land use intensity on the degree of specialization of 72 plant-herbivore networks, including effects mediated by the increase in the proportion of exotic plant species. Contrary to our expectation, the net effect of land use intensity on network specialization was positive. However, this positive effect of land use intensity was partially canceled by an opposite effect of the proportion of exotic plant species on network specialization. When we analyzed networks composed exclusively of endophagous herbivores separately from those composed exclusively of exophagous herbivores, we found that only endophages showed a consistent change in network specialization at higher land use levels. Altogether, these results indicate that land use intensity is an important ecological driver of network specialization, by way of reducing the local host range of herbivore guilds with highly specialized feeding habits. However, because the effect of land use intensity is offset by an opposite effect owing to the proportion of exotic host species, the net effect of land use in a given herbivore assemblage will likely depend on the extent of the replacement of native host species with exotic ones.

## Introduction

Consumer-resource interactions between herbivorous insects and their host plants represent a large fraction of terrestrial food webs. In pristine habitats, plant-herbivore networks are mostly characterized by highly specialized interactions, since most insect species consume only a small subset of the total set of host plant species [1–2]. Given the accelerated rates of habitat conversion into human-modified environments, it is important to understand whether interaction specialization in plant-herbivore networks increases or decreases with land use intensification. There is a broad consensus that habitat alteration by human activities has negative consequences on native species, usually reducing their overall diversity [3–6]. The anthropogenic modification of natural habitats also favors the establishment of invasive exotic species, which may outcompete native plants [7–9]. Species extinctions and the replacement of native species with exotic ones can disrupt coevolutionary interactions, causing community-wide changes in species interaction networks [10–12]. Therefore, the effect of land use intensity on ecological interaction networks, if any, is potentially modified by the concomitant increase in the proportion of exotic species in human-altered environments.

Because many herbivorous insects have a high degree of feeding specialization [13–15], the loss of native plant species can lead to the local extinction of more specialized herbivores. In addition, because herbivores depend on their host plants to maintain their populations, feeding specialization (i.e., narrow host range) constrains the successful colonization of new habitats by herbivores and, consequently, the expansion of their geographical distribution. Thus, as land use intensifies, the average feeding specialization of herbivorous insects is expected to drop, as more generalist herbivores tend to persist longer than specialists do [16–17].

Exotic plant species, in general, have little shared evolutionary history with native insects, thus making them less likely to be consumed by specialized native herbivores when compared with native plants [18]. Hence, exotic plant species would be mostly consumed by a subset of generalist herbivore species that also feed on many native plant species [19]. An increase in the relative availability of exotic plants could lead to a reduction in the overall specialization of plant-herbivore interactions owing to an increase in the average proportion of host plant species used by the remaining herbivorous insect fauna dominated by generalist species. Therefore, an increase in the replacement of native host species with exotic species is expected to reinforce the negative effect of land use intensity on the degree of specialization in local interaction networks between plants and their herbivores.

An important feature of herbivorous insects is the way in which they feed on their host plants. Perhaps the most distinctive dichotomy is between endophages and exophages. Endophagous herbivores feed and often develop concealed within plant tissues (e.g., gall makers and leaf miners). In contrast, exophages are free-living herbivores that feed externally on plant tissues (e.g., leaf chewers and phloem suckers). Exophages have been shown to be more generalist, in terms of host range, than endophages [20–21]. The higher specialization levels of endophages in comparison with exophages are explained by the greater interaction intimacy with their hosts, which requires specialized adaptations to develop inside plant organs and to deal with plant defenses [20]. Since specialized herbivores are more prone to local extinction due to the replacement of native by exotic host plants, plant-herbivore networks made up of endophagous herbivores are expected to be more sensitive to higher proportions of exotic host plants than exophage-plant networks.

**Figure 1:**
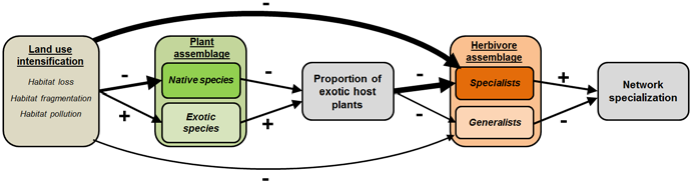
A conceptual model for the effects of land use intensification on the specialization of interactions in plant-herbivore networks. First, human land use is expected to have a negative impact on the species richness of native host species, while facilitating the invasion and establishment of exotic plant species. Consequently, plant assemblages under higher levels of land use intensity are predicted to have a higher proportion of exotic plant species. Both land use intensification and the increase in the proportion of exotic host plant species would promote the loss of herbivore species at the local scale, but with greater negative impacts on the specialist herbivores than on generalists. Because of the greater reduction in the number of specialist herbivorous species, the proportion of monophagous herbivores is predicted to decrease, whereas the network connectance is predicted to increase with land use intensification and higher proportions of exotic host plant species.

In this study, we retrieved plant-herbivore networks surveyed under different levels of land use intensification and with distinct proportions of exotic host species, to investigate the extent to which human alteration of habitats leads to predictable changes in the specialization of plant-herbivore interactions. Because most plant-herbivore studies provided only binary (i.e., presence-absence) interaction records, we used network connectance and proportion of monophagous herbivores as proxy variables for network specialization. Connectance is the proportion of possible interactions that are realized (i.e., recorded) in a network. In plant-herbivore networks, connectance can be interpreted as the mean proportion of host plant species used by the herbivores as well as the mean number of herbivorous species recorded on each plant species. Monophagous herbivores are the insect species recorded on a single host plant species and thus represent the highest level of feeding specialization. Specifically, we addressed the following questions: 1) Do higher levels of human land use and higher proportions of exotic host species lead to consistent changes in the connectance of plant-herbivore networks? 2) Are monophagous herbivores more sensitive to the effects of land use intensity and proportion of exotic host species? 3) Do plant-herbivore networks composed of internal- versus external-plant feeders respond differently to the increase in land use intensity and in the proportion of exotic host species? We outline the expected effects of land use intensification and proportion of exotic host plant species in a conceptual model (Fig. 1). Connectance and proportion of monophages are respectively inversely and directly related to the overall specialization of interactions. Therefore, we predict positive effects of land use intensity on connectance and negative effects on the proportion of monophages. Using a large spectrum of plant-herbivore networks in terms of phylogenetic groups and types of environments, this study aims to provide a broad assessment of the community-wide effects of land use intensification on plant-herbivore networks.

## Material and Methods

### Plant-herbivore networks

We produced a comprehensive compilation of sets of local interaction records between host plants and herbivorous insects (S1 and S2 Tables). We used Scopus and Google Scholar to search for studies reporting local plant-herbivore interaction networks using the following keywords: (plant*) and (herbivor*) and (network* or interaction* or web*) and (survey* or list*). In addition, we inspected the Interaction Web Database of the National Center for Ecological Analysis and Synthesis (www.nceas.ucsb.edu/interactionweb) and the cited literature in some key review studies on plant-insect interactions [21–24]. We also included available data from MSc and PhD theses, research reports, and reports from government agencies.

The following criteria were applied to include a plant-herbivore network in our analyses: (1) The original study should provide at least a basic description of the study area to allow categorization of land use intensity; (2) herbivores were recorded on their host plants; (3) all plants should be potential hosts for any herbivorous insect; (4) the network should contain at least five plant and five insect species, totaling at least 10 species; and (5) at least 70% of the herbivores should be identified at the species or genus level.

### Land use intensity

In order to assess the degree of habitat alteration to which each plant-herbivore network was subjected, we developed an index of land use intensity. Because most of the compiled studies did not include a detailed description of land use or human-induced modifications that might allow a fine classification of land use intensity, we used a simple classification scheme based on the descriptions of the sampling sites in the original studies. We defined the following four categories of land use intensity: (1) pristine natural habitats (e.g., primary forests); (2) natural habitats moderately impacted by human activities (e.g., secondary forests); (3) agricultural or ruderal habitats with high diversity of plant species (e.g., agroforestry systems and abandoned pastures); and (4) agricultural or urban habitats with low diversity of plant species (e.g., monocultures and urban orchards). This categorization of land use was used as an ordinal variable ranging from 1 to 4.

### Exotic plants

For each network, we classified the plant species as native or exotic according to plant databases available for the country where the network was studied (S3 Table). This binary classification of plants according to their origin can be interpreted as a distinction between plants with some (native) or little (exotic) shared evolutionary history with the native host species of herbivorous insects. Plants that were not identified to the species level in the original studies could not be checked for their origin, so we assumed, conservatively, that they were natives (less than 14% of the plant species). No distinction between native and exotic herbivores was made; we assumed that in most networks the large majority of insect species are native to the sampled region. After classifying each plant species in each network as native or exotic, we calculated the proportion of exotic plant species in each plant-herbivore network. To meet normality requirements, we applied the arcsine transformation to the proportion of exotic plants when necessary.

### Taxonomic variables

When describing plant-herbivore networks, authors often choose to limit the sampling procedure within a taxon. For example, some networks include only plants from a given family [19], or only insects from a given order [25]. We used the taxonomic span of the sampled plant set and of the herbivorous insects as explanatory variables for network specialization measures. For plant taxonomic levels we used the following categories: Family = 1, Suborder = 2, Order = 3, Superorder = 4, Subclass = 5, Class = 6, Subdivision = 7, Division = 8. For herbivorous insects, the taxonomic levels were Subfamily = 1, Family = 2, Suborder = 3, Order = 4, Subclass = 5, Class = 6.

The taxonomic spans of plants and insects are necessarily related to the phylogenetic diversity of the plant and insect assemblages, respectively. Because of the difficulty to obtain well-resolved phylogenies for insects and plants, we used taxonomic diversity as surrogate of phylogenetic diversity of the plant-insect networks. For this, we used the index Average Taxonomic Distinctness (AvTD), which is based on topological distances between species in the taxonomic tree [26]. This index can be interpreted as the expected number of nodes between any two randomly selected species in the considered community [26]. Schweiger et al. [27] compared different phylogenetic diversity indices and recommended the use of AvTD because it is unbiased by species richness and reflects phylogeny per se. To calculate the AvTD, we used six taxonomic levels for plants (genus, subfamily, family, order, superorder, and subclass) and five taxonomic levels for insects (genus, subfamily, family, superfamily, and order). We used PRIMER 6 software to calculate the AvTD [28].

### Estimating network specialization

We used network connectance and the proportion of monophagous herbivores as proxies for network specialization. Connectance is, in fact, an inverse measure of overall interaction specialization because, in networks with higher connectance, species tend to have more connections (i.e., to be less specialized); thus, the higher the connectance, the lower the specialization of plant-herbivore networks. The connectance was calculated as the residuals from a linear regression between the number of realized interactions and the number of potential interactions (both log-transformed) across plant-herbivore networks (hereafter “residual connectance”). By using the residual connectance, we controlled for the negative relationship between network size (i.e., total number of plant and insect species) and connectance, a well-known issue when comparing distinct networks [29]. Residual connectance therefore allows the comparison of different-sized networks in terms of higher or lower connectance than expected based on their size (positive and negative residuals, respectively) [24].

The proportion of monophages (i.e., insects consuming a single plant species) in each network was used as a further measure of network specialization. Note that we measured specialization at the local scale, so that a monophagous species in a given plant-herbivore network may be a generalist herbivore if all its populations are considered [30]. In addition, the proportion of monophages is a good measure of network fragility at the local scale because the coextinction of each monophagous herbivore is caused by the extinction of a single host species.

### Data analysis

We used a path analysis approach [31] to quantify the effects of land use intensity and the proportion of exotic host species on the residual connectance and the proportion of monophages in plant-herbivore networks, while controlling for the confounding effects of plant and herbivore taxonomic span and the average taxonomic distinctness of both groups. The path analysis for residual connectance was based on the following rationale (Fig. 1): (1) the index of land use intensity is the explanatory variable of interest and was included in all models as an exogenous variable; (2) the proportion of exotic plants is expected to be positively influenced by land use intensity and to have a positive effect on the residual connectance, being included in all models as both an exogenous and endogenous variable; (3) plant taxonomic span and herbivore taxonomic span are explanatory variables for the residual connectance, for the proportion of exotic plants, and for the AvTD of plants and insects, and both were included as explanatory variables to control for their possible confounding effects on the relationships between the variables of interest; (4) the AvTD of plants is expected to be influenced by plant taxonomic span, the proportion of exotic plants, and land use intensity, and to affect residual connectance, being thus included in the models as both exogenous and endogenous variables; and (5) the AvTD of insects is expected to be influenced by herbivore taxonomic span and by the AvTD of plants and all variables that also potentially influence the AvTD of plants. We used similar models to determine the effects of explanatory variables on the proportion of monophages, and to construct models of the residual connectance and proportion of monophages separately for endophagous and exophagous herbivores.

The model fits were assessed through chi-squared tests and by examination of the Tucker-Lewis Fit Index (TLI), the Comparative Fit Index (CFI), and the Root Mean Square Error of Approximation (RMSEA). Statistical power was calculated using the approach introduced by MacCallum et al. [32] and the R code developed by Preacher and Coffman [33]. Normality was checked using the Mardia test, and outliers were inspected using the Mahalanobis distance. Path analyses were built and fitted in AMOS version 5.0 [34].

## Results

**Figure 2:**
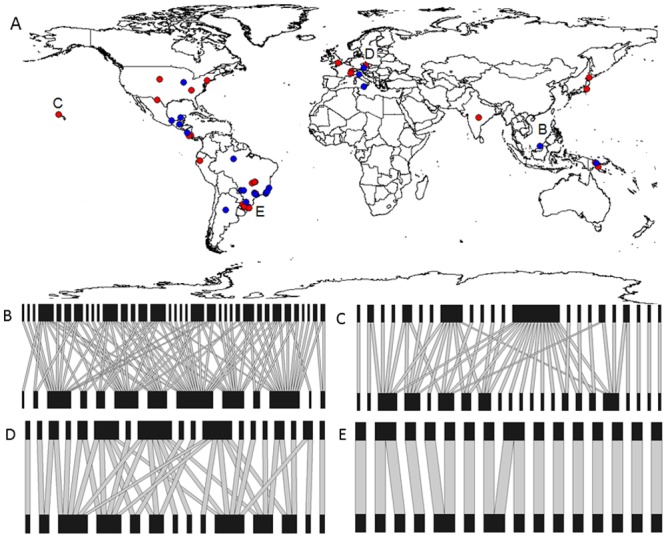
Global distribution of the (A) 72 community-wide plant-herbivore networks used in the current study. Some of the networks localities are indistinguishable at this map scale. Blue plots represent endophagous networks and red plots represent exophagous networks. (B-E) Examples of plant-herbivore networks with different levels of land use intensity (LUI) and specialization. Herbivores are on the top and plants on the bottom of the networks. The networks are arranged in descending order of residual connectance. (B) Network of Nakagawa et al. [45] localized in habitat with LUI level 1; (C) Network of Henneman and Memmot [46] localized in habitat with LUI level 2; (D) Network of Masetti et al. [47] localized in habitat with LUI level 3; (E) Network of Santos et al. [48] localized in habitat with LUI level 4.

We selected the 72 plant-herbivore networks that meet the five inclusion criteria used in this study (see Methods). These networks belong to several ecoregions distributed across the globe (Fig. 2) and comprise a wide variety of taxonomic and functional groups of herbivorous insects, including leaf chewers, stem chewers, phloem suckers, flower head feeders, fruit-flies, leaf miners and gall makers (S1 and S2 Tables). The mean local richnesses of herbivores and plants were 23.5 (± 21.3 SD) and 24.1 (± 23.1 SD), respectively. We found 33 plant-herbivore networks made up of endophagous herbivores, and 39 with exophagous herbivores.

**Figure 3:**
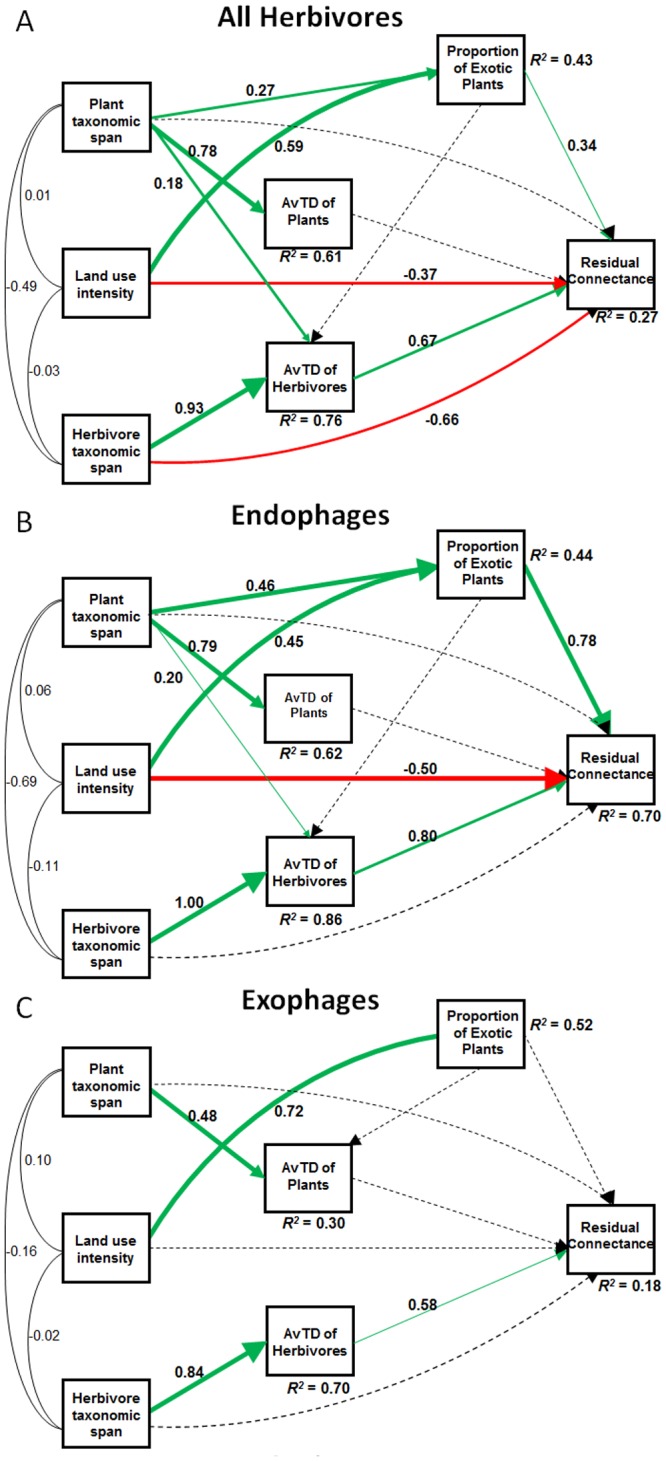
Path analyses of residual connectance in plant-insect networks of (A) all herbivores, (B) endophages, and (C) exophages, explained by land use intensity, proportion of exotic plants, plant taxonomic span, herbivore taxonomic span, and average taxonomic distinctness (AvTD) of plants and of insects. Numbers on paths between variables are standardized path coefficients (scaled by the standard deviations of the variables). Green arrows represent positive effects and red arrows represent negative effects. The thickness of lines and arrows is proportional to effect size.

**Figure 4:**
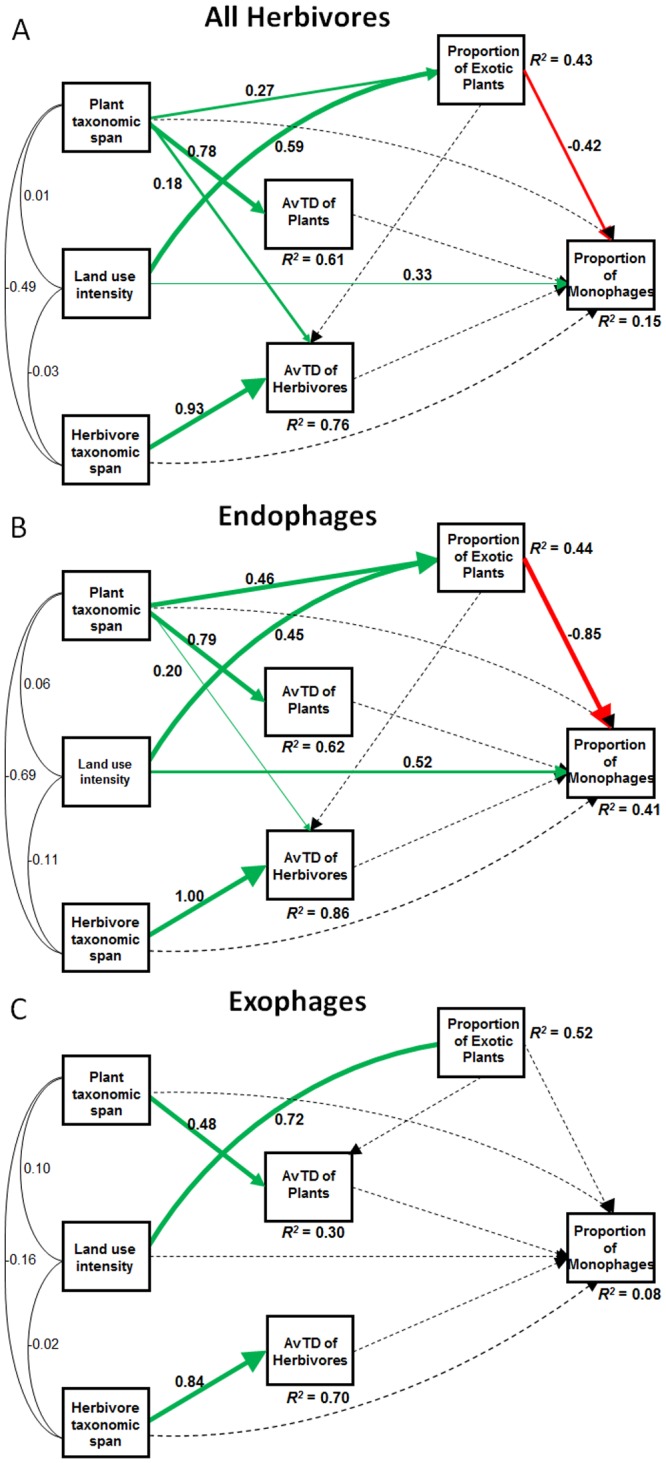
Path analyses of proportion of monophages from plant-insect networks of (A) all herbivores, (B) endophages, and (C) exophages. For details and explanations, see legend for Fig. 3.

The path models explained 27%, 70% and 18% of the variation in residual connectance considering all networks, only plant-endophage networks and only plant- exophage networks, respectively (Fig. 3). The three path models showed good fit to the data (Table 1). The models also explained 15% (all networks), 41% (endophages only), and 8% (exophages only) of the variation (Fig. 4) in the proportion of monophagous herbivores.

**Table 1:** Statistical values of fit, power and explanation of path models for the residual connectance and proportion of monophages.

**Response variables**	**Path models**	**Model fit**				Statistical power*
		**χ^2^ (p-value)**	**CFI**	**TLI**	**RMSEA**	
Residual connectance	All herbivores	6.00 (0.422)	1.000	0.999	0.004	> 0.999
	Endophages	4.17 (0.653)	1.000	1.045	< 0.001	> 0.999
	Exophages	3.03 (0.932)	1.000	1.174	< 0.001	0.988
Proportion of monophages	All herbivores	6.00 (0.422)	1.000	0.999	0.004	> 0.999
	Endophages	4.17 (0.654)	1.000	1.047	< 0.001	> 0.999
	Exophages	3.03 (0.932)	1.000	1.184	< 0.001	0.982

TLI: Tucker-Lewis Fit Index; CFI: Comparative Fit Index; RMSEA: Root Mean Square Error of Approximation. Statistical power was calculated using the approach by MacCallum et al. [32].

We found that the total effect of land use intensity on the residual connectance is partly offset by the proportion of exotic host plant species (Table 2; Fig. 3). Contrary to our expectations, the direct effect of land use intensity on residual connectance was negative, indicating that the overall specialization in plant-herbivore networks increases under higher levels of anthropogenic alteration of natural habitats. On the other hand, we found a positive effect of the proportion of exotic host plants on residual connectance. The combination of the direct and the indirect (i.e., mediated by other variables) effects of land use intensity resulted in a net negative impact on the residual connectance (Table 2), and thus a positive effect on overall feeding specialization. When endophage- and exophage-plant networks were analyzed separately, we found significant effects of land use intensity and proportion of exotic host species only for endophages (Fig. 3). Residual connectance was also positively affected by the AvTD of herbivores in all models, which was highly influenced by the taxonomic span of the sampled herbivores (Fig. 3).

**Table 2:** Explanatory models for the residual connectance according to path analyses in Fig. 3 with direct and indirect coefficients and their relative contributions to the explained variation (R^2^), following Lewinsohn [49].

**Residual connectance**	**Explanatory variables**	**Correlation (*r*)**	**Effect**		
			**Direct (*d*)**	**Indirect (*i*)**	**Total (*e* = *d* + *i*)**
All herbivores	Land use intensity	-0.19	-0.36	0.17	-0.19
	Proportion of exotic plants	0.01	0.35	-0.06	0.29
	AvTD of herbivores	0.22	0.66	-	0.66
	Plant taxonomic span	-0.14	-0.18	0.04	-0.14
	Herbivore taxonomic span	0.09	-0.61	0.62	0.01
Endophages	Land use intensity	-0.21	-0.50	0.30	-0.20
	Proportion of exotic plants	0.13	0.78	-0.10	0.68
	AvTD of herbivores	0.29	0.79	-	0.79
	Plant taxonomic span	-0.30	-0.48	0.24	-0.24
	Herbivore taxonomic span	0.26	-0.72	0.80	0.08
Exophages	AvTD of herbivores	0.22	0.57	-	0.57
	Herbivore taxonomic span	0.06	-0.45	0.48	0.03

Only variables with significant total effects are presented. The “direct effect” (*d*) expresses how much a given variable changes in response to changes in another variable while controlling for the effect of all other variables in the model. The ‘‘indirect effect’’ (*i*) expresses the influence of a given variable on another variable that is mediated by one or more variables through causal relationships presented in the model.

We found similar results when the proportion of monophagous herbivores was used as a measure of interaction specialization in plant-herbivore networks (Fig. 4). Land use intensity and the proportion of exotic host species showed opposite effects on the proportion of monophagous herbivore species, but these effects did not hold for plant-herbivore networks composed by exophagous insects (Fig. 4; Table 3). The direct effect of land use intensity on the proportion of monophagous herbivore species was strongly positive, while the effect mediated by other variables was weakly negative, resulting in a positive net effect (Table 3).

**Table 3:** Explanatory models for the proportion of monophages according to path analyses in Fig. 4 with direct and indirect coefficients and their relative contributions to the explained variation (R^2^).

**Proportion of monophages**	**Explanatory variables**	**Correlation (*r*)**	**Effect**		
			**Direct (*d*)**	**Indirect (*i*)**	**Total (*e* = *d* + *i*)**
All herbivores	Land use intensity	0.08	0.32	-0.22	0.10
	Proportion of exotic plants	-0.17	-0.41	0.03	-0.38
	Plant taxonomic span	-0.01	0.09	-0.03	0.06
Endophages	Land use intensity	0.15	0.51	-0.35	0.16
	Proportion of exotic plants	-0.40	-0.85	0.05	-0.80
	Plant taxonomic span	-0.05	0.35	-0.39	-0.04

Only variables with significant total effects are presented. The “direct effect” (*d*) expresses how much a given variable changes in response to changes in another variable while controlling for the effect of all other variables in the model. The ‘‘indirect effect’’ (*i*) expresses the influence of a given variable on another variable that is mediated by one or more variables through causal relationships presented in the model.

## Discussion

### Do land use intensity and exotic host plants affect the degree of specialization in plant-herbivore interactions?

Our results show that land use intensification and an increasing proportion of exotic host plant species have opposite effects on the overall interaction specialization of plant-herbivore networks. Contrary to our expectations, land use intensity has a direct negative effect on network connectance and a positive effect on the proportion of monophagous herbivores. The term “direct effect” here means an effect that is not mediated by other variables in the path models, but that can be mediated by non-measured variables. Conversely, connectance increases and the proportion of monophagous herbivores decreases as the proportion of exotic host species increases. Since the proportion of exotic host species tends to increase at higher levels of land use intensity [7–8], the positive direct effect of land use on network specialization is somewhat counterbalanced by the negative effect of exotic host species. Consequently, the overall net effect of land use intensity on the specialization of plant-herbivore interactions is only moderately positive (Tables 2 and 3).

It is possible that network specialization increases with habitat changes, because more specialized herbivorous insects tend to be associated with more resilient plant species [35]. Thus, although specialists would be especially vulnerable to losing their hosts, the resilience of their hosts to human-induced changes might explain their persistence even in strongly altered habitats where many generalists have been lost. In addition, monophages can be more metabolically and behaviorally efficient than generalists in exploiting their host plants [30].

Another explanation for the increase in network specialization in highly impacted habitats could be that, at the local scale, generalist insects tend to become specialized in a few plant species after habitat conversion by human activities. In fact, spatial and temporal variation in the use of host plants might cause herbivores to have few local host species [15, 30]. Although we did not include total richness of native plants (including the non-host plant species) in the models (because that information was not available in the compiled studies), we have strong expectations that it should decrease in more intensively modified habitats. Thus, it is possible that specialists in habitats with high land use intensity are not true specialist insects, but generalists constrained to be local specialists due to the lack of alternative food resources [15]. We therefore suggest that the modifications promoted by land use, through the reduction in the number of potential native host plant species, might restrict the diet of the herbivores and enhance the occurrence of local specialists.

From the standpoint of herbivorous insects, an increase in network specialization can be interpreted as a decline in the number of alternative host-plants, which increases the probability of secondary extinctions of herbivores following the loss of host species [12, 25]. This might be one of the mechanisms by which extreme levels of habitat conversion (as in croplands and pastures) greatly increase the vulnerability of host-parasitoid networks [36]. In plant-herbivore networks, previous results show a positive relationship between high network connectance (i.e., decreased specialization) and increased herbivore persistence [37, 38].

Land use intensity also has a negative indirect effect on specialization mediated by an increase in the proportion of exotic plants. An explanation for our results is that the herbivore fauna associated with exotic plants is restricted, composed of few species [19] that are usually highly polyphagous [39]. An expected effect of exotic plants on the structure of plant-herbivore networks is the replacement of specialist herbivores with generalists [40]. Herbivorous species that have a restricted host range are more sensitive to exotic plant invasion [8]. As a result of the increasing richness and abundance of exotic plants, the richness of more specialized herbivores diminishes rapidly, but the number of interactions diminishes more slowly, because the loss of each specialist removes few interactions. Therefore, the number of realized interactions diminishes much more slowly than the number of potential interactions. This process of disproportional loss of herbivore specialists leads to a net reduction of specialization in plant-herbivore networks.

### Discrepant effects on plant-herbivore networks composed by endophages vs. exophages

Our findings provide evidence that external and internal plant feeders have different responses to land use intensity. In the present study, we found that land use intensity has direct and indirect effects on the community-wide specialization of endophagous insects, but little or no effect on the community-wide specialization of exophagous insects.

Endophages tend to be more specialized than exophages [20, 41], and thus, they have a higher degree of dependence on a smaller set of host species [21, 42]. Furthermore, because they develop inside the plant during their immature stages, endophages are better protected than exophages against external microclimatic changes caused by human action. For those reasons, it is expected that the effects of land use intensity on endophages occur mainly via changes in host plant composition. The replacement of native host species with exotic ones has a greater impact on endophagous insects, as some exophages can colonize the novel hosts more rapidly [21].

We recently showed that an increase in the proportion of exotic plants has a greater impact on the diversity of endophagous than on the diversity of exophagous herbivore insects [40]. We also observed this pattern in the current study, as land use intensity had significant effects only on those networks composed of endophagous insects. These insects are most affected because exotic plant species often lead to a decline in the populations of native plants, including species that were dominant before the invasion [9]. For generalist herbivores, this effect might be buffered by relatively broad diets, allowing them to tolerate the loss or reduction of important host species by compensating or switching to alternative hosts.

### How does the sampled taxonomic range affect network specialization in plant-herbivore networks?

Several studies on plant-herbivore interaction networks have shown that interactions between species are phylogenetically compartmentalized [2, 43]. Phylogenetically related plants tend to be consumed by phylogenetically related insect species, such as folivorous Lepidoptera in the Brazilian Cerrado [2]. Researchers usually set the taxonomic range of the studied network arbitrarily, and the diversity of recorded interactions is likely to depend on the taxonomic inclusiveness of both plants and insects. However, we are not aware of previous studies that investigated whether the choice of range of taxonomic groups of plants or insects influences estimates of specialization in plant-insect networks.

Our results show that estimates of network specialization are negatively affected by herbivore taxonomic range. Thus, networks studied over a broader taxonomic range (i.e., class or order) are likely to have a smaller proportion of realized interactions than networks that span a more restricted taxonomic unit (i.e., family or genus). This can be explained by the fact that interaction connectivity within a given network compartment is greater than interaction connectivity between the different compartments [44]. The sampling of more inclusive taxonomic levels leads to an increase in the number of compartments in the networks, each of them only weakly linked to the others.

Phylogenetic diversity (as measured by AvTD) of herbivores also influenced network residual connectance; however, the effect was positive. This might occur because phylogenetically diverse assemblages are more likely to contain super-generalist herbivores. Additionally, when sampling a wider taxonomic span of herbivores (e.g., orders) in phylogenetically diverse assemblages, researchers are likely to sample a smaller fraction of the diversity of these taxa, with the overrepresentation of generalist species leading to higher perceived network specialization.

## Conclusions

Human-induced modifications of natural habitats have promoted the rapid loss of biological diversity, mainly through changes in the diversity of species and their interactions. Our study indicates that land use intensity is an important driver of network specialization, reducing the local host range of herbivore guilds already characterized by highly specialized feeding habits. However, because the effect of land use intensity is also offset by an opposite effect of the proportion of exotic host species, the net overall effect will depend on the extent of the replacement of native with exotic host plant species. Our findings also point to different effects on assemblages of endophagous or exophagous herbivores, so that only plant-herbivore networks of endophagous insects show a change in interaction specialization due to increases both in land use intensity and in the proportion of exotic host species

## Supporting Information

S1 Table References to the 72 plant-insect networks used in this study. In the case of repeated references the same study contained more than one network.
(DOCX)

S2 Table Descriptions of the insect-plant interaction networks used in the analysis of the effects of land use intensity on the herbivores insect diversity. List of assemblage characteristics related to insect diversity and geographical aspects. The codes of the networks correspond to references listed in S1 Table.
(DOCX)

S3 Table List of online databases and sources used in the determination of exotic and native plant status. (DOCX)
