# Supplementary material for: Contrasting Effects of Land Use Intensity and Exotic Host Plants on the Specialization of Interactions in Plant-Herbivore Networks
Source: PLoS One. 2015 Jan 7;10(1):e0115606. doi: 10.1371/journal.pone.0115606 (PMC4286214; doi:10.1371/journal.pone.0115606)
Supplement: S1 Table — In the case of repeated references the same study contained more than one network. (DOCX) [file pone.0115606.s001.docx]

Table S1. References to the 72 plant-insect networks used in this study. In the case of repeated references the same study contained more than one network.

| **Code** | **Reference** |
| --- | --- |
| 1 | Joern, A. 1979. Feeding patterns in grasshoppers (Orthoptera: Acrididae): factors influencing diet specialization. Oecologia 38: 325-347. |
| 2 | Joern, A. 1979. Feeding patterns in grasshoppers (Orthoptera: Acrididae): factors influencing diet specialization. Oecologia 38: 325-347. |
| 3 | Joern, A. 1985. Grasshopper dietary (Orthoptera: Acrididae) from a Nebraska Sand Hills Prairie. Transaction of the Nebraska Academy of Sciences 13: 21-32. |
| 4 | Santos. J.P.; Soglio. F.K.D. & Redaelli. L.R. 2006. Plantas hospedeiras de dípteros minadores em pomar de citros em Montenegro, RS. Arquivos do Instituto de Biologia 73: 235-241. |
| 5 | Delfino, M.A. & Buffa, L.M. 2008. Afidos en plantas ornamentales de Córdoba, Argentina (Hemiptera: Aphididae). Neotropical Entomology 37: 74-80. |
| 6 | Favret, C.; Duggan, J.J.; Sanders, N.J. & Phill, L.R. 2010. Actual and inferred checklist of the aphids (Hemiptera: Aphididae) of the Great Smoky Mountains National Park, with attendant ant and host plant associations. Proceedings of the Entomological Society of Washington 112: 381-403. |
| 7 | Peronti, A.L.B.G. & Sousa-Silva, C.R. 2002. Aphids (Hemiptera: Aphidoidea) of ornamental plants from São Carlos, São Paulo state, Brazil. Revista de Biología Tropical 50: 137-144. |
| 8 | Lethmayer, C. 1998. Occurrence of aphids in an agricultural area with sown weed strips. In: Nieto Nafría. J.M. & Dixon. A.F.G. (Eds.). Aphids in natural and managed ecosystems. Universidad de Léon. Léon. Spain. Pp. 601-608. |
| 9 | Müller, C.B.; Adriannse, I.C.T.; Belshaw, R. & Godfray, H.C.J. 1999. The structure of an aphid-parasitoid community. Journal of Animal Ecology 68: 346-370. |
| 10 | Starý, P. & Havelka, J. 2008. Fauna and associations of aphid parasitoids in an up-dated farmland area (Czech Republic). Bulletin of Insectology 61: 251-276. |
| 11 | Starý, P. & Havelka, J. 2008. Fauna and associations of aphid parasitoids in an up-dated farmland area (Czech Republic). Bulletin of Insectology 61: 251-276. |
| 12 | Starý, P. & Havelka, J. 2008. Fauna and associations of aphid parasitoids in an up-dated farmland area (Czech Republic). Bulletin of Insectology 61: 251-276. |
| 13 | Starý, P. & Havelka, J. 2008. Fauna and associations of aphid parasitoids in an up-dated farmland area (Czech Republic). Bulletin of Insectology 61: 251-276. |
| 14 | Van Veen, F.J.F.; Müller, C.B.; Pell, J.K. & Godfray, H.C.J. 2008. Food web structure of three guilds of natural enemies: predators, parasitoids and pathogens of aphids. Journal of Animal Ecology 77: 191-200. |
| 15 | García-Robledo, C.; Erickson DL.; Staines CL.; Erwin TL & Kress WJ. 2013. Tropical Plant–Herbivore Networks: Reconstructing Species Interactions Using DNA Barcodes. PLoS ONE 8(1): e52967. doi:10.1371/journal.pone.0052967 |
| 16 | Basset, Y. & Samuelson, G.A. 1996. Ecological characteristics of an arboreal community of Chrysomelidae in Papua New Guinea. In: Chrysomelidae Biology: Ecological Studies. Amsterdam. Academic Publishing. pp. 243-262. |
| 17 | Pokon, R.; Novotny, V. & Samuelson, G.A. 2005. Host specialization and species richness of root-feeding chrysomelid larvae (Chrysomelidae, Coleoptera) in a New Guinea rain forest. Journal of Tropical Ecology 21: 595-604. |
| 18 | Janzen, D.H. 1980. Specificity of seed-attacking beetles in a Costa Rican deciduous forest. Journal of Ecology 68: 929-952. |
| 19 | Thum, A.B. & Costa, E.C. 1997. Coreidae (Heteroptera) associados a espécies florestais. Ciência Florestal 7: 27-31. |
| 20 | Almeida-Neto, M. 2006. Efeito da degradação de habitat sobre a estrutura de interações entre plantas e insetos fitófagos. PHD Thesis, Campinas, Brazil. |
| 21 | Almeida-Neto, M. 2006. Efeito da degradação de habitat sobre a estrutura de interações entre plantas e insetos fitófagos. PHD Thesis, Campinas, Brazil. |
| 22 | Almeida-Neto, M. 2006. Efeito da degradação de habitat sobre a estrutura de interações entre plantas e insetos fitófagos. PHD Thesis, Campinas, Brazil. |
| 23 | Nascimento, A.R. 2010. Estudo de interações de parasitoides de insetos endófagos em frutos do Cerrado. Master Thesis, São Carlos, Brazil. |
| 24 | Carregaro, J.B. 2011. Insetos associados a botões florais de plantas do Cerrado. PHD Thesis, Brasília, Brazil. |
| 25 | Perre, P.; Loyola, R.D.; Lewinsohn, T.M. & Almeida-Neto, M. 2011. Insects on urban plants: contrasting the flower head feeding assemblages on native and exotic hosts. Urban Ecosystem 14: 711-722. |
| 26 | Kollár, J. 2011. Gall-inducing arthropods associated with ornamental woody plants in a City Park of Nitra (SW Slovakia). Acta entomologica serbica 16: 115-126. |
| 27 | Ibanez, S.; Lavorel, S.; Puijalon, S. & Moretti, M. 2013. Herbivory mediated by coupling between biomechanical traits of plants and grasshoppers. Functional Ecology 27:479-489. |
| 28 | Rathcke, B.J. 1976. Competition and coexistence with a guild of herbivorous insects. Ecology 57: 76-87. |
| 29 | Garcia, A.H. 1999. Levantamento, identificação e avaliação dos danos de insetos em árvores ornamentais na área urbana de Goiânia (GO). Pesquisa Agropecuária Tropical 29: 77-81. |
| 30 | Henneman, M.L. & Memmot, J. 2001. Infiltration of a Hawaiian community by introduced biological control agents. Science 293: 1314-1316. |
| 31 | Henneman, M.L. & Memmot, J. 2001. Infiltration of a Hawaiian community by introduced biological control agents. Science 293: 1314-1316. |
| 32 | Nakagawa, M.; Itioka, T.; Momose, K.; Yumoto, T.; Komai, F.; Morimoto, K.; Jordal, B.H.; Kato, M.; Kaliang, H.; Hamid, A.A.; Inoue, T. & Nakashizuka, T. 2003. Resource use of insect seed predators during general flowering and seeding events in a Bornean dipterocarp rain forest. Bulletin of Entomological Research 93: 455-466. |
| 33 | Nakagawa, M.; Itioka, T.; Momose, K.; Yumoto, T.; Komai, F.; Morimoto, K.; Jordal, B.H.; Kato, M.; Kaliang, H.; Hamid, A.A.; Inoue, T. & Nakashizuka, T. 2003. Resource use of insect seed predators during general flowering and seeding events in a Bornean dipterocarp rain forest. Bulletin of Entomological Research 93: 455-466. |
| 34 | Otway, S.J.; Hector, A. & Lawton, J.H. 2005. Resource dilution effects on specialist insect herbivores in a grassland biodiversity experiment. Journal of Animal Ecology 74: 234-240. |
| 35 | Weiblen, G.D.; Webb, C.O.; Novotny, V.; Basset, Y. & Miller, S.E. 2006. Phylogenetic dispersion of host use in a tropical insect herbivore community. Ecology 87: 62-75. |
| 36 | Massa, B.; Rizzo, M.C. & Caleca, V. 2001. Natural alternative hosts of Eulophidae (Hymenoptera: Chalcidoidea) parasitoids of the citrus leafminer *Phyllocnistis citrella* Stainton (Lepidoptera: Gracillariidae) in the Mediterranean Basin. Journal of Hymenoptera Research 10: 91-100. |
| 37 | Valladares, G. & Salvo, A. 2001. Community dynamics of leafminers (Diptera: Agromyzidae) and their parasitoids (Hymneoptera) in a natural habitat from Central Argentina. Acta Oecologica 22: 301-309. |
| 38 | Masetti, A.; Lanzoni, A.; Burgio, G. & Süss, L. 2004. Faunistic study of the Agromyzidae (Diptera) on weeds of marginal areas in Northern Italy Agroecosystems. Annals of Entomological Society of America 97: 1252-1262. |
| 39 | Masetti, A.; Lanzoni, A.; Burgio, G. & Süss, L. 2004. Faunistic study of the Agromyzidae (Diptera) on weeds of marginal areas in Northern Italy Agroecosystems. Annals of Entomological Society of America 97: 1252-1262. |
| 40 | Masetti, A.; Lanzoni, A.; Burgio, G. & Süss, L. 2004. Faunistic study of the Agromyzidae (Diptera) on weeds of marginal areas in Northern Italy Agroecosystems. Annals of Entomological Society of America 97: 1252-1262. |
| 41 | Santos, J.P.; Redaelli, L.R.; Soglio, F.K.D.; Foelkel, E. & Costa. V.A. 2009. Variação sazonal de lepidópteros minadores e seus parasitóides em plantas de crescimento espontâneo em pomar orgânico de citros em Montenegro, RS, Brasil. Arquivos do Instituto de Biologia 76: 381-391. |
| 42 | Embrapa. 2007. Avaliação ecológica de riscos de algodoeiro resistente a insetos: levantamento e seleção de lepidópteros não-alvo. Boletim de Pesquisa e Desenvolvimento 202: 1-17. |
| 43 | Diniz, I.R.; Morais, H.C.; Botelho, A.M.F.; Venturoli, F. & Cabral, B.C. 1999. Lepidopteran caterpillar fauna on lactiferous host plants in the Central Brazilian Cerrado. Revista Brasileira de Biologia 59: 627-635. |
| 44 | Kan, E.; Fukuhara, N. & Hidaka, T. 2003. Parasitism by tachinid parasitoids (Diptera: Tachinidae) in connection with their survival strategy. Applied Entomology and Zoology 38: 131-140. |
| 45 | Monteiro, R.F.; Macedo, M.V.; Nascimento, M.S. & Cury, R.S.F. 2007. Composição, abundância e notas sobre a ecologia de larvas de lepidópteros associados a cinco espécies de plantas hospedeiras no Parque Nacional da Restinga de Jurubatiba. RJ. Revista Brasileira de Entomologia 51: 476-483. |
| 46 | Hirao, T. & Murakami, M. 2008. Quantitative food webs of lepidopteran leafminers and their parasitoids in a Japanese deciduous forest. Ecological Research 23: 159-168. |
| 47 | Bodner, F.; Brehm, G.; Homeier, J.; Strutzenberger, P. & Fiedler, K. 2010. Caterpillars and host plant records for 59 species of Geometridae (Lepidoptera) from a montane rainforest in Southern Ecuador. Journal of Insect Science 10: 1-22. |
| 48 | Tiple, A.D.; Khurad, A.M. & Dennis, R.L.H. 2011. Butterfly larval host plant use in a tropical urban context: Life history associations, herbivory, and landscape factors. Journal of Insect Science 11:65 available online: insectscience.org/11.65 |
| 49 | Silva, N.A.P. 2011. Biologia de lagartas de Lycaenidae em inflorescências de plantas no cerrado do Brasil Central. Master Thesis, Brasília, Brasil. |
| 50 | Lopes, B.C. 1995. Treehoppers (Homoptera. Membracidae) in Southeastern Brazil: use of host plants. Revista Brasileira de Zoologia 12: 595-608. |
| 51 | Wallace, M.S. 2008. Occurrence of treehoppers (Hemiptera: Membracidae: Smiliinae) on oaks in Delaware Water Gap National Recreation Area. 2004–2006. Journal of Insect Science 8: 1-16. |
| 52 | Lopes. O.J.; Link. D. & Basso. L.V. 1974. Pentatomídeos de Santa Maria – lista preliminar de plantas hospedeiras. Revista Centro Ciências Rurais 4: 317-322. |
| 53 | Costa, E.C.; Bogorni, P.C. & Bellomo, V.H. 1995. Percevejos coletados em copas de diferentes espécies florestais. Pentatomidae-1. Ciência Florestal Santa Maria 5: 123-128. |
| 54 | Garlet, J.; Roman, M. & Costa, E.C. 2010. Pentatomídeos (Hemiptera) associados a espécies nativas em Itaara. RS. Brasil. Biotemas 23: 91-96. |
| 55 | Hernández-Ortiz, V.; Pérez-Alonso, R. & Wharton, R.A. 1994. Native parasitoids associated with the genus *Anastrepha* (Dipt.: Thephritidae) in Los Tuxtlas, Veracruz, Mexico. Entomophaga 39: 171-178. |
| 56 | Borge, M.N.R. & Basedow, T. 1997. A survey on the occurrence and flight periods of fruit fly species (Diptera: Tephritidae) in a fruit growing area in southwest Nicaragua. 1994/95. Bulletin of Entomological Research 87: 405-412. |
| 57 | Aluja, M.; Rull, J.; Sivinski, J.; Norrbom, A.L.; Wharton, R.A.; Macias-Ordóñez, R.; Díaz-Fleischer, F. & López, M. 2003. Fruit flies of the genus Anastrepha (Diptera: Tephritidae) and associated native parasitoids (Hymenoptera) in the tropical tainforest Biosphere Reserve of Montes Azules, Chiapas, Mexico. Environmental Entomology 32: 1377-1385. |
| 58 | Uchôa-Fernandes, M.A.; Molina, R.M.S.; Oliveira, I.; Zucchi, R.A.; Canal, N.A. & Diaz, N.B. 2003. Larval endoparasitoids (Hymenoptera) of frugivorous flies (Diptera, Tephritoidea) reared from fruits of the cerrado of the State of Mato Grosso do Sul, Brazil. Revista Brasileira de Entomologia 47: 181-186. |
| 59 | Uchôa-Fernandes, M.A.; Molina, R.M.S.; Oliveira, I.; Zucchi, R.A.; Canal, N.A. & Diaz, N.B. 2003. Larval endoparasitoids (Hymenoptera) of frugivorous flies (Diptera, Tephritoidea) reared from fruits of the cerrado of the State of Mato Grosso do Sul, Brazil. Revista Brasileira de Entomologia 47: 181-186. |
| 60 | Uchôa-Fernandes, M.A.; Molina, R.M.S.; Oliveira, I.; Zucchi, R.A.; Canal, N.A. & Diaz, N.B. 2003. Larval endoparasitoids (Hymenoptera) of frugivorous flies (Diptera, Tephritoidea) reared from fruits of the cerrado of the State of Mato Grosso do Sul, Brazil. Revista Brasileira de Entomologia 47: 181-186. |
| 61 | Uramoto, K.; Walder, J.M.M. & Zucchi, R.A. 2004. Biodiversidade de moscas-das-frutas do gênero *Anastrepha* (Diptera. Tephritidae) no *campus* da ESALQ-USP, Piracicaba, São Paulo. Revista Brasileira de Entomologia 48: 409-414. |
| 62 | Novotny, V.; Clarke, A.R.; Drew, R.A.I. & Balagawi, S. & Clifford, B. 2005. Host specialization and species richness of fruit flies (Diptera: Tephritidae) in a New Guinea rain forest. Journal of Tropical Ecology 21: 67-77. |
| 63 | Hernandez-Ortiz, V.; Delfín-González, H.; Escalante-Tio, A. & Manrique-Saide, P. 2006. Hymenoptera parasitoids of *Anastrepha* fruit flies (Diptera: Tephritidae) reared from different hosts in Yukatan. Mexico. The Florida Entomologist 89: 508-515. |
| 64 | Leal, M.R. 2008. Dinâmica populacional das moscas-das-frutas (Diptera: Tephritidae) e introdução de *Diachasmimorpha longicaudata* Ashmead (Hymenoptera: Braconidae) para controle da praga na região norte do estado do Rio de Janeiro. Master Thesis, Seropédica, Brazil. |
| 65 | Leal, M.R. 2008. Dinâmica populacional das moscas-das-frutas (Diptera: Tephritidae) e introdução de *Diachasmimorpha longicaudata* Ashmead (Hymenoptera: Braconidae) para controle da praga na região norte do estado do Rio de Janeiro. Master Thesis. Seropédica. Brazil. |
| 66 | Uramoto, K.; Martins, D.S. & Zucchi, R.A. 2008. Fruit flies (Diptera. Tephritidae) and their associations with native host plants in a remnant area of the highly endangered Atlantic Rain Forest in the State of Espírito Santo. Brazil. Bulletin of Entomological Research 98: 457-466. |
| 67 | Costa, S.G.M.; Querino, R.B.; Ronchi-Teles, B.; Penteado-Dias, A.M.M. & Zucchi, R.A. 2009. Parasitoid diversity (Hymenoptera: Braconidae and Figitidae) on frugivorous larvae (Diptera: Tephritidae and Lonchaeidae) at Adolpho Ducke Forest Reserve, Central Amazon Region, Manaus, Brazil. Brazilian Journal of Biology 69: 363-370. |
| 68 | Leal, R.M.; Souza, S.A.S.; Aguiar-Menezes, E.L.; Lima-Fiolho, M. & Menezes, E.B. 2009. Diversidade de moscas-das-frutas, suas plantas hospedeiras e seus parasitoides nas regiões Norte e Noroeste do Estado do Rio de Janeiro, Brasil. Ciência Rural 39: 627-634. |
| 69 | Ronchi-Teles, B.; Dutra, V.S.; Costa, A.P.T.; Aguiar-Menezes, E.L.; Mesquita, A.C.A. & Silva, J.G. 2011. Natural host plants and native parasitoids associated with *Anastrepha pulchra* and other *Anastrepha* species (Diptera: Tephritidae) in Central Amazon, Brazil. The Florida Entomologist 94: 347-349. |
| 70 | Sabedot-Bordin, S.M.; Bogus, G.M.; Bampi, D. & Garcia, F.R.M. 2011. Tefritídeos endófagos (Diptera: Tephritidae) associados à Asteraceae em Chapecó, Santa Catarina. Biotemas 24: 15-20. |
| 71 | Cavalleri, A. 2005. Comunidades de Tripes (Insecta: Thysanoptera) em flores e ramos, com ênfase em Asteraceae, no Parque Estadual de Itapuã, Viamão, RS. Master Thesis, Porto Alegre, Brazil. |
| 72 | Pinent, S.M.J.; Romanowski, H.P.; Redaelli, L.R. & Cavalleri, A. 2011. Thysanoptera: plantas visitadas e hospedeiras no Parque Estadual de Itapuã, Viamão, RS. Brasil. Iheringia 95: 9-16. |
