# Supplementary material for: Contrasting Effects of Land Use Intensity and Exotic Host Plants on the Specialization of Interactions in Plant-Herbivore Networks
Source: PLoS One. 2015 Jan 7;10(1):e0115606. doi: 10.1371/journal.pone.0115606 (PMC4286214; doi:10.1371/journal.pone.0115606)
Supplement: S2 Table — List of assemblage characteristics related to insect diversity and geographical aspects. The codes of the networks correspond to references listed in S1 Table. (DOCX) [file pone.0115606.s002.docx]

Table S2. Descriptions of the insect-plant interaction networks used in the analysis of the effects of land use intensity on the herbivores insect diversity. List of assemblage characteristics related to insect diversity and geographical aspects. The codes of the networks correspond to references listed in Table S1.

| **Code** | **Herbivore guild** | **Herbivore taxon** | **Land use intensity** | **Latitude** | **Longitude** | **Altitude (m)** | **Biome** | **Ecoregion** | **Ecozone** | **Country** |
| --- | --- | --- | --- | --- | --- | --- | --- | --- | --- | --- |
| 1 | Exophagous | Acrididae | 2 | 30.000 | -103.000 | 1416 | TGSS | Nebraska Sand Hills mixed grasslands | Nearctic | United States of America |
| 2 | Exophagous | Acrididae | 1 | 30.000 | -103.000 | 1416 | TGSS | Nebraska Sand Hills mixed grasslands | Nearctic | United States of America |
| 3 | Exophagous | Acrididae | 2 | 41.566 | -101.700 | 1117 | TGSS | Texas blackland prairies | Nearctic | United States of America |
| 4 | Endophagous | Agromyzidae | 4 | -29.983 | -51.767 | 40 | TSMBF | Araucaria moist forest | Neotropic | Brazil |
| 5 | Exophagous | Aphididae | 3 | -37.872 | -4.784 | 474 | TSGSS | Córdoba montane savanna | Neotropic | Argentina |
| 6 | Exophagous | Aphididae | 2 | 35.683 | -83.533 | 267 | TBMF | Appalachian-Blue Ridge forests | Nearctic | United States of America |
| 7 | Exophagous | Aphidoidea | 4 | -22.015 | -47.891 | 830 | TSGSS | Cerrado | Neotropic | Brazil |
| 8 | Exophagous | Aphididae | 4 | 46.000 | 7.000 | 556 | TBMF | Western European broadleaf forests | Palearctic | Switzerland |
| 9 | Exophagous | Aphididae | 2 | 51.000 | -0.641 | 68 | TBMF | North Atlantic moist mixed forests | Palearctic | United Kingdon |
| 10 | Exophagous | Aphididae | 2 | 49.513 | 14.931 | 537 | TBMF | Western European broadleaf forests | Palearctic | Czech Republic |
| 11 | Exophagous | Aphididae | 4 | 49.513 | 14.931 | 537 | TBMF | Western European broadleaf forests | Palearctic | Czech Republic |
| 12 | Exophagous | Aphididae | 4 | 49.513 | 14.931 | 537 | TBMF | Western European broadleaf forests | Palearctic | Czech Republic |
| 13 | Exophagous | Aphididae | 3 | 49.513 | 14.931 | 537 | TBMF | Western European broadleaf forests | Palearctic | Czech Republic |
| 14 | Exophagous | Aphididae | 2 | 51.000 | -0.641 | 68 | TBMF | North Atlantic moist mixed forests | Palearctic | United Kingdon |
| 15 | Exophagous | Cassidinae | 1 | 10.422 | -84.015 | 83 | TSDBF | Central American dry forests | Neotropic | Costa Rica |
| 16 | Exophagous | Chrysomelidae | 2 | -7.400 | 146.733 | 1300 | TSMBF | Central Range montane rain forests | Australasia | New Guinea |
| 17 | Exophagous | Chrysomelidae | 2 | -5.233 | 145.683 | 200 | TSMBF | Northern New Guinea lowland rain and freshwater swamp forests | Australasia | New Guinea |
| 18 | Exophagous | Coleoptera | 1 | 10.000 | -85.000 | 283 | TSDBF | Central American dry forests | Neotropic | Costa Rica |
| 19 | Exophagous | Coreidae | 1 | -28.272 | 54.659 | 178 | TSMBF | Araucaria moist forest | Neotropic | Brazil |
| 20 | Endophagous | Insecta | 4 | -22.006 | -47.832 | 760 | TSGSS | Cerrado | Neotropic | Brazil |
| 21 | Endophagous | Insecta | 4 | -22.260 | -47.785 | 830 | TSGSS | Cerrado | Neotropic | Brazil |
| 22 | Endophagous | Insecta | 4 | -22.280 | -47.821 | 700 | TSGSS | Cerrado | Neotropic | Brazil |
| 23 | Endophagous | Insecta | 1 | -21.967 | -47.869 | 890 | TSGSS | Cerrado | Neotropic | Brazil |
| 24 | Endophagous | Insecta | 1 | -15.916 | -47.916 | 1061 | TSGSS | Cerrado | Neotropic | Brazil |
| 25 | Endophagous | Insecta | 4 | -22.000 | -47.069 | 876 | TSGSS | Cerrado | Neotropic | Brazil |
| 26 | Endophagous | Insecta | 4 | 48.000 | 14.000 | 140 | TBMF | Pannonian mixed forests | Palearctic | Slovak |
| 27 | Exophagous | Orthoptera | 2 | 6.400 | 45.033 | 555 | TCF | Alps conifer and mixed forests | Palearctic | France |
| 28 | Endophagous | Insecta | 1 | 40.000 | -88.000 | 223 | TGSS | Central forest-grasslands transition | Nearctic | United States of America |
| 29 | Exophagous | Insecta | 4 | -16.000 | -49.000 | 792 | TSGSS | Cerrado | Neotropic | Brazil |
| 30 | Exophagous | Lepidoptera | 2 | 22.000 | -159.000 | 771 | TSMBF | Hawaii tropical moist forests | Oceania | Hawaii, USA |
| 31 | Exophagous | Lepidoptera | 1 | 22.000 | -159.000 | 771 | TSMBF | Hawaii tropical moist forests | Oceania | Hawaii, USA |
| 32 | Endophagous | Insecta | 1 | 4.333 | 113.833 | 60 | TSMBF | Borneo lowland rain forests | Indo-Malay | Malaysia |
| 33 | Endophagous | Insecta | 1 | 4.333 | 113.833 | 60 | TSMBF | Borneo lowland rain forests | Indo-Malay | Malaysia |
| 34 | Exophagous | Insecta | 2 | 51.000 | -0.641 | 68 | TBMF | North Atlantic moist mixed forests | Palearctic | United Kingdon |
| 35 | Exophagous | Insecta | 2 | -5.000 | 145 | 200 | TSMBF | Northern New Guinea lowland rain and freshwater swamp forests | Australasia | New Guinea |
| 36 | Endophagous | Insecta | 3 | 37.000 | 14 | 660 | MFWS | South Apennine mixed montane forests | Palearctic | Italy |
| 37 | Endophagous | Agromyzidae | 2 | -31.333 | -64.167 | 800 | TSGSS | Córdoba montane savanna | Neotropic | Argentina |
| 38 | Endophagous | Agromyzidae | 3 | 45.000 | 11.000 | 62 | TBMF | Po Basin mixed forests | Palearctic | Italy |
| 39 | Endophagous | Agromyzidae | 3 | 45.000 | 11.000 | 62 | TBMF | Po Basin mixed forests | Palearctic | Italy |
| 40 | Endophagous | Agromyzidae | 3 | 45.000 | 11.000 | 62 | TBMF | Po Basin mixed forests | Palearctic | Italy |
| 41 | Endophagous | Lepidoptera | 4 | -29.983 | -51.767 | 40 | TSMBF | Araucaria moist forest | Neotropic | Brazil |
| 42 | Exophagous | Lepidoptera | 4 | -15.817 | -47.567 | 1075 | TSGSS | Cerrado | Neotropic | Brazil |
| 43 | Exophagous | Lepidoptera | 1 | -15.917 | -47.883 | 1076 | TSGSS | Cerrado | Neotropic | Brazil |
| 44 | Exophagous | Lepidoptera | 3 | 36.000 | 140 | 75 | TSMBF | Nansei islands subtropical evergreen forests | Palearctic | Japan |
| 45 | Exophagous | Lepidoptera | 1 | -22.000 | -41.000 | 8 | TSMBF | Atlantic Coast restingas | Neotropic | Brazil |
| 46 | Exophagous | Lepidoptera | 2 | 42.717 | 141.600 | 90 | TBMF | Hokkaido deciduous forests | Palearctic | Japan |
| 47 | Exophagous | Lepidoptera | 2 | -3.967 | -79.083 | 2100 | MGS | Cordillera Central páramo | Neotropic | Ecuador |
| 48 | Exophagous | Lepidoptera | 3 | -30.983 | 79.150 | 319 | TSDBF | Chhota-Nagpur dry deciduous forests | Indo-Malay | India |
| 49 | Exophagous | Lycaenidae | 1 | -15.917 | -47.917 | 1050 | TSGSS | Cerrado | Neotropic | Brazil |
| 50 | Exophagous | Membracidae | 2 | -22.188 | -47.119 | 640 | TSGSS | Cerrado | Neotropic | Brazil |
| 51 | Exophagous | Membracidae | 2 | 41.100 | -74.983 | 327 | TBMF | Appalachian-Blue Ridge forests | Nearctic | United States of America |
| 52 | Exophagous | Pentatomidae | 4 | -29.683 | -53.783 | 131 | TSMBF | Araucaria moist forest | Neotropic | Brazil |
| 53 | Exophagous | Pentatomidae | 3 | -30.000 | -53.000 | 115 | TSMBF | Araucaria moist forest | Neotropic | Brazil |
| 54 | Exophagous | Pentatomidae | 2 | -29.610 | -53.765 | 425 | TSMBF | Araucaria moist forest | Neotropic | Brazil |
| 55 | Endophagous | Tephritidae | 2 | 18.567 | -95.067 | 530 | TSMBF | Petén-Veracruz moist forests | Neotropic | Mexico |
| 56 | Endophagous | Tephritidae | 3 | 11.000 | -86.000 | 445 | TSMBF | Central American montane forests | Neotropic | Nicaragua |
| 57 | Endophagous | Tephritidae | 1 | 16.000 | -90.500 | 140 | TSMBF | Chiapas montane forests | Neotropic | Mexico |
| 58 | Endophagous | Tephritidae | 4 | -22.708 | -47.633 | 546 | TSGSS | Cerrado | Neotropic | Brazil |
| 59 | Endophagous | Tephritidae | 2 | -5.137 | 145.686 | 200 | TSMBF | Northern New Guinea lowland rain and freshwater swamp forests | Australasia | New Guinea |
| 60 | Endophagous | Tephritidae | 4 | 20.300 | -89.700 | 38 | TSMBF | Yucatán moist forests | Neotropic | Mexico |
| 61 | Endophagous | Tephritidae | 4 | -21.800 | -41.300 | 9 | TSMBF | Atlantic Coast restingas | Neotropic | Brazil |
| 62 | Endophagous | Tephritidae | 4 | -21.300 | -40.933 | 10 | TSMBF | Atlantic Coast restingas | Neotropic | Brazil |
| 63 | Endophagous | Tephritidae | 2 | -19.100 | -39.750 | 60 | TSMBF | Alto Paraná Atlantic forests | Neotropic | Brazil |
| 64 | Endophagous | Tephritidae | 1 | -2.883 | -59.983 | 98 | TSMBF | Southwest Amazon moist forests | Neotropic | Brazil |
| 65 | Endophagous | Tephritidae | 4 | -21.300 | -40.933 | 10 | TSMBF | Atlantic Coast restingas | Neotropic | Brazil |
| 66 | Endophagous | Tephritidae | 1 | -2.883 | -59.983 | 98 | TSMBF | Southwest Amazon moist forests | Neotropic | Brazil |
| 67 | Endophagous | Tephritidae | 4 | -27.096 | -52.618 | 660 | TSMBF | Araucaria moist forest | Neotropic | Brazil |
| 68 | Endophagous | Tephritoidea | 3 | -20.527 | -55.837 | 170 | TSGSS | Cerrado | Neotropic | Brazil |
| 69 | Endophagous | Tephritoidea | 3 | -20.650 | -55.331 | 173 | TSGSS | Cerrado | Neotropic | Brazil |
| 70 | Endophagous | Tephritoidea | 3 | -20.437 | -54.082 | 308 | TSGSS | Cerrado | Neotropic | Brazil |
| 71 | Exophagous | Thysanoptera | 2 | -30.450 | -51.080 | 129 | TSMBF | Araucaria moist forest | Neotropic | Brazil |
| 72 | Exophagous | Thysanoptera | 2 | -30.367 | -51.033 | 20 | TSMBF | Araucaria moist forest | Neotropic | Brazil |

Legend: MFWS = Mediterranean forests, woodlands, and shrub; MGS = Montane grasslands and shrublands; TBMF = Temperate broadleaf and mixed forests; TCF = Temperate coniferous forests; TGSS = Temperate grasslands, savannas, and shrublands; TSDBF = Tropical and subtropical dry broadleaf forests; TSGSS = Tropical and subtropical grasslands, savannas, and shrublands; TSMBF = Tropical and subtropical moist broadleaf forests
