# Supplementary material for: Contrasting Effects of Land Use Intensity and Exotic Host Plants on the Specialization of Interactions in Plant-Herbivore Networks
Source: PLoS One. 2015 Jan 7;10(1):e0115606. doi: 10.1371/journal.pone.0115606 (PMC4286214; doi:10.1371/journal.pone.0115606)
Supplement: S3 Table — (DOCX) [file pone.0115606.s003.docx]

Table S3. List of online databases and sources used in the determination of exotic and native plant status.

| **Country** | **Online database or Source** | **Access date** |
| --- | --- | --- |
| Argentina | <http://www.floraargentina.edu.ar/espsin.asp> | 22-Apr-13 |
| Brazil | <http://floradobrasil.jbrj.gov.br/jabot/listaBrasil/PrincipalUC/PrincipalUC.do> | 17-Apr-13 |
| Costa Rica | <http://darnis.inbio.ac.cr/ubis/FMPro?-db=UBI&-lay=Weball&-format=findmore.html&-view> | 20-Apr-13 |
| Ecuador | <http://www.tropicos.org/ProjectAdvSearch.aspx?projectid=2> | 20-Apr-13 |
| Europe | <http://rbg-web2.rbge.org.uk/FE/fe.html#instructions> | 21-Apr-13 |
| Europe | <http://wbd.etibioinformatics.nl/bis/flora.php?menuentry=soorten> | 21-Apr-13 |
| Europe | <http://www.europe-aliens.org/speciesSearch.do> | 21-Apr-13 |
| Hawaii | <http://plants.usda.gov/du/DistributionUpdate.html> | 19-Apr-13 |
| Italy | <http://dbiodbs1.univ.trieste.it/checklist/index.php?procedure=search> | 20-Apr-13 |
| Japan | <http://foj.c.u-tokyo.ac.jp/gbif/foj/> | 21-Apr-13 |
| Malasya | <http://chm-malaysia.org/Bio-Diversity-Databases/Flora-Database.aspx> | 21-Apr-13 |
| Mexico | http://www.conabio.gob.mx/invasoras/index.php/Especies_invasoras_-_Plantas | 20-Apr-13 |
| Nicaragua | <http://www.tropicos.org/NameSearch.aspx?projectid=7> | 20-Apr-13 |
| Panama | <http://www.tropicos.org/NameSearch.aspx?projectid=56> | 20-Apr-13 |
| Papua New Guinea | <http://www.pngplants.org/PNGCensus/index_copy.html> | 20-Apr-13 |
| Swiss | <http://www.infoflora.ch/> | 21-Apr-13 |
| UK | <http://www.brc.ac.uk/plantatlas/index.php?q=title_page> | 21-Apr-13 |
| USA | <http://plants.usda.gov/du/DistributionUpdate.html> | 19-Apr-13 |
| Various | <http://www.gisin.org/cwis438/websites/GISINDirectory/SpeciesStatus_Result.php?WebSiteID=4> | 21-Apr-13 |
| Various | <http://www.iucnredlist.org/> | 21-Apr-13 |
| Central America | CITES (2010) Updated listings of fauna and flora species. Included in the CITES Appendices Distributed in Central America and Dominican Republic. CITES, Geneva | 20-Apr-13 |
| Czech Republic | Pysek P, S adlo J and Mandák B (2003c) Alien ﬂora of the Czech Republic, its composition, structure and history. In: Child LE, et al (eds) Plant Invasions: Biological Threats and Management Options, pp 113–130. Backhuys Publishers, Leiden, The Netherlands | 21-Apr-13 |
| Czech Republic | Sadlo J, Chytrý M, & Pyšek P (2007) Regional species pools of vascular plants in habitats of the Czech Republic. Preslia 79:303-321 | 21-Apr-13 |
| Czech Republic | Pyšek P, et al. (2012) Catalogue of alien plants of the Czech Republic (2nd edition): checklist update, taxonomic diversity and invasion patterns. Preslia 84: 155–255 | 21-Apr-13 |
| Europe | Daisie (2008) The Handbook of Alien Species in Europe. Springer, Berlin | 21-Apr-13 |
| Europe | **Scalera R**, Genovesi P, Essl F, Rabitsch W, 2012. [The impacts of invasive alien species in Europe](http://www.eea.europa.eu/publications/impacts-of-invasive-alien-species). EEA Technical report no.16/2012. | 21-Apr-13 |
| India | Reddy CS (2008) Catalogue of invasive alien flora of India. Life Science Journal 5:84-89 | 9-May-13 |
| Italy | Celesti-Grapow et al. (2010) Flora vascolare alloctona e invasiva delle regioni d'Italia | 21-Apr-13 |
| Mexico | Williams JK (2010) Additions to the alien vascular flora of Mexico, with comments on the shared species of Texas, Mexico, and Belize. Phytoneuron 3: 1-7 | 20-Apr-13 |
| Mexico | Villaseñor, J. L. y Espinosa-García, F.J. (2004). The alien flowering plants of Mexico. Diversity and Distributions 10: 113-12 | 20-Apr-13 |
| Slovakia | Jarolímek I. (2012): Inventory of the alien flora of Slovakia. Preslia 84: 257–309 | 21-Apr-13 |
